# Juggling confidentiality and safety: a qualitative study of how general practice clinicians document domestic violence in families with children

**DOI:** 10.3399/bjgp17X689353

**Published:** 2017-01-31

**Authors:** Jessica Drinkwater, Nicky Stanley, Eszter Szilassy, Cath Larkins, Marianne Hester, Gene Feder

**Affiliations:** Leeds Institute of Health Sciences, Leeds.; School of Social Work, Care and Community, University of Central Lancashire, Preston.; Centre for Academic Primary Care, School of Social and Community Medicine, University of Bristol, Bristol.; School of Social Work, Care and Community, University of Central Lancashire, Preston.; Professor of gender, violence, and international policy, School for Policy Studies, University of Bristol, Bristol.; Centre for Academic Primary Care, School of Social and Community Medicine, University of Bristol, Bristol.

**Keywords:** child safeguarding, domestic violence, electronic patient record, general practice, primary care, qualitative research

## Abstract

**Background:**

Domestic violence and abuse (DVA) and child safeguarding are interlinked problems, impacting on all family members. Documenting in electronic patient records (EPRs) is an important part of managing these families. Current evidence and guidance, however, treats DVA and child safeguarding separately. This does not reflect the complexity clinicians face when documenting both issues in one family.

**Aim:**

To explore how and why general practice clinicians document DVA in families with children.

**Design and setting:**

A qualitative interview study using vignettes with GPs and practice nurses (PNs) in England.

**Method:**

Semi-structured telephone interviews with 54 clinicians (42 GPs and 12 PNs) were conducted across six sites in England. Data were analysed thematically using a coding frame incorporating concepts from the literature and emerging themes.

**Results:**

Most clinicians recognised DVA and its impact on child safeguarding, but struggled to work out the best way to document it. They described tensions among the different roles of the EPR: a legal document; providing continuity of care; information sharing to improve safety; and a patient-owned record. This led to strategies to hide information, so that it was only available to other clinicians.

**Conclusion:**

Managing DVA in families with children is complex and challenging for general practice clinicians. National integrated guidance is urgently needed regarding how clinicians should manage the competing roles of the EPR, while maintaining safety of the whole family, especially in the context of online EPRs and patient access.

## INTRODUCTION

Domestic violence and abuse (DVA) and child safeguarding are internationally recognised as interlinked problems, impacting on the health and wellbeing of all family members.^1^^–^4 In the UK, general practice is often the first point of access to health services for families experiencing DVA and clinicians have equal duty of care for all family members.^5^^,^^6^ Responding to DVA in families with children is complex and involves management of different family members, and coordination of a multidisciplinary response.^6^^,^^7^ Research and serious case reviews have identified that the general practice response is often lacking.^5^^,^^8^^–^10 Both the World Health Organization (WHO) and the National Institute for Health and Care Excellence (NICE) have called for better documentation of DVA.^11^^,^^12^

In UK general practice, documenting takes place in the electronic patient record (EPR) using a combination of national (Read) diagnostic and procedure codes and free text. Research and health policy conceptualises EPRs as a mechanism to improve health care through increased efficiency, influence professional behaviour, and empower patients through access to their records.^13^^–^15 Documenting in EPRs is a key mechanism for information sharing in child safeguarding.^6^^,^^7^ UK policy encourages health professionals to document child safeguarding concerns in all family members’ records, recognising the need for a whole-family approach.^7^^,^^16^ This is supported by national guidance on the mechanism of documenting (including which codes to use) and reinforced through mandatory training for general practice clinicians.^16^^,^^17^ However, there is increasing recognition of unintended consequences of EPRs and their impact on individual and organisational behaviour.^18^^–^20 Specifically, there are ongoing professional concerns about documenting stigmatising information, including DVA.^21^ With DVA, confidentiality is essential given the risk of abuse escalation when a perpetrator discovers disclosure. From April 2016, patients in the UK have the right to request access to their EPR online and this has intensified fears about coercion and breaches in confidentiality in relation to DVA.^22^

The complex reality faced by general practice clinicians managing DVA in families with children is not recognised; existing evidence and guidance focus on child safeguarding and DVA separately. The present study reports a nested qualitative study (RESPONDS: Researching Education to Strengthen Primary care ON Domestic violence and Safeguarding) of general practice clinicians’ response to DVA, in particular how and why clinicians document DVA in families.^23^

**How this fits in**

Domestic violence and abuse (DVA) and child safeguarding are interlinked problems, but this is not necessarily recognised in evidence, policy, or professional guidance. Focusing on how clinicians document DVA in families with children reveals variation and inconsistency in practice. This is partly because of the multiple different roles of the electronic patient record. National integrated guidance is urgently needed to help clinicians to support all family members, especially in light of the move to online patient access.

## METHOD

### Study design

This was a qualitative study using semi-structured telephone interviews.

### Sampling and recruitment

GPs and practice nurses (PNs) were recruited from general practices in six areas of England. Areas were selected to represent high and low levels of specialist DVA service provision in the north, south, and Midlands, including metropolitan, urban, and semi-rural locations. Sampling was based on geographical spread and recruitment continued until saturation of new themes was achieved.^24^

### Data collection and analysis

Data collection was by semi-structured telephone interviews lasting on average half an hour. Participants were sent an information leaflet, consent form, and vignette that depicted a patient (‘Sarah’) disclosing physical violence from her partner (‘Danny’) to a GP or PN. She also described his controlling behaviour towards their three children aged 7, 5, and 2 years. The vignette allowed for exploration of clinicians’ views on responding to DVA and child safeguarding even where their own experience was limited. Additional questions included how clinicians respond to the different family members, what they would document, and what actions they would take following disclosure. Interviews were audiotaped with consent, and transcribed verbatim.

Transcripts were loaded into qualitative data analysis software (NVivo) and analysed thematically.^25^ A coding framework was developed through reading and re-reading transcripts by a multidisciplinary team with different methodological backgrounds, taking a constant comparison approach. This framework was influenced by concepts in the literature and recommendations from separate professional and service user expert panels.

## RESULTS

Fifty-four clinicians (42 GPs and 12 PNs) took part in semi-structured telephone interviews. Demographics of participants are reported in Table 1. This study focused on interviewees’ responses about documenting as this illuminates the current organisational and attitudinal barriers faced by general practice clinicians managing DVA in families. Themes identified were knowledge and attitudes about documenting DVA in families; the role of the EPR; and tensions between the different roles of the EPR.

**Table 1.** Demographic details of research participants

	**GPs**	**Nurses**
**Sex**		
Male	17	0
Female	25	12

**Age range, years**		
21–34	8	2
35–44	11	0
45–54	15	8
55–64	5	1
Not known	3	1

**Experience managing domestic violence and abuse, number of cases**		
>5	5	0
A few	13	1
1	0	2
None	18	8
None, but aware of case at surgery	6	1

### Knowledge and attitudes regarding documentation

Participants described various methods of documenting DVA and child safeguarding in the EPR from national codes to free-text entries (Box 1). Many clinicians were unconfident in documenting DVA. This is consistent with only half of the clinicians having any experience of DVA, few having any DVA training, and that there is no national guidance on which codes should be used. Nine clinicians (six GPs and three nurses) admitted that they did not know how to document DVA. One nurse said that recording DVA should be the doctor’s role:

*‘I’d have to speak to the doctors to, you know, work out how, what we would do about that* [the case in the vignette]*, whether that was something that would need to go on the children’s notes, in which case really they* [the doctor] *should put that on.’*

(PN 07, female, age 45–54 years)

Box 1. **Electronic mechanisms used by general practice clinicians for documenting domestic violence and abuse in families with children**

**Table:** 

**Coding mechanisms**	**Examples**
Read Codes^a^ within the EPR	Mainly use child safeguarding Read Codes: ‘26 different codes’ Little use of existing domestic violence and abuse Read Codes Read Code as ‘cause for concern’ Read Code as ‘depression’ Practice has own template of Read Codes
Hidden alerts within EPR software	Alert message on home screen Reminders on home screen Practice wide code word Individual GP code ‘marital problems ???’ Safeguarding icon Traffic light system
Messaging systems	Internal messaging system — audit trail — not in notes External NHS email to all partners/clinicians — not in notes Liaising with OOHs
Free text	Detailed free-text comments — document injuries Use the patients’ own words Vague free-text comments

a *NHS Digital defines Read Codes as follows: ‘* Read Codes are a coded thesaurus of clinical terms and have been used in the NHS since 1985 … They provide the standard vocabulary by which clinicians can record patient findings and procedures in health and social care IT systems across primary and secondary care.’ *26 EPR = electronic patient record. OOHs = out of hours.*

Clinicians decisions’ about documenting DVA were shaped by attitudes about naming different forms of DVA and when to document these in the notes:

*‘Violence in the home I think is one* [Read Code] *we use, if we know that Mum has been hit and presumably we could use that because he* [Danny in vignette] *has punched her, so that, that’s the commonest one I tend to end up using but it’s not so easy if it’s just name calling or emotional abuse.’*

(GP 25, female, 45–54 years)

This was reflected in a wide variety of responses to how to document the DVA in the vignette:

*‘I suppose at the end if, you know, it* [the case] *was as clear as this* [the vignette]*, then my, my problem title* [Read Code] *would be “domestic violence.”’*

(GP 37, female, 45–54 years)

*‘I think with this lady* [Sarah in vignette] *I would, I might, I’d probably put the problem title* [Read Code] *more to do with “depression”… as a clinical diagnosis but in the … comment section or in the history section* [free text] *… I would be writing what I saw as significant issues.’*

(GP 42, male, 45–54 years)

Clinicians were more confident about how to document child safeguarding. This is unsurprising, given that all responders reported some mandatory child safeguarding training which often covers how to document, including which codes to use. Despite this, six clinicians were not aware of a practice-wide documenting policy for child safeguarding and nine admitted to ‘doing their own thing’:

‘*To be honest we haven’t had this discussion, I’m not actually sure we have a practice policy.’*

(GP 01, female, 35–44 years)

Where practices did have policies, there was wide variation at a national and local level. In one study site, a GP stated that they used and regularly updated the national codes:

‘Yes we have certain codes that we use and then these are updated from social services.’

(GP 26, female, 25–34 years)

At another practice in the same area, they reported developing their own practice policy with no input from Children’s Services.

Even when clinicians were confident in their knowledge about documenting, inconsistency still occurred because of the different roles of the EPR.

### Role of the EPR

Four roles of the EPR were described when documenting DVA in families with children: a legal document; providing continuity of care; information sharing to improve safety; and a patient-owned record.

#### A legal document

Some clinicians discussed documenting in the victim’s EPR to make a legal record of injuries and abuse. These clinicians described the EPR as a ‘factual record’ (GP 13, female, 45–54 years). Their strategies for documenting DVA reflected this:

*‘I would record the, the reason for, for presenting* [using a code] *and then in a sort of free text … I would record the actual nature of this disclosure, what the disclosure was, whether there were any hard confirmatory signs of physical injury, you know, sort of bruising, size and shape, location, that sort of thing, and then I would record my sort of immediate actions and what my sort of next steps would be in terms of where I was going to take it in terms of follow-up.’*

(GP 34, female, 25–34 years)

Some of these clinicians had concerns about documenting in other family members’ EPRs events for which they only had the victim’s account:

‘So I would be reluctant to put in somebody’s notes that they were perpetrating domestic violence because of course it’s not proven, but I would perhaps put a code in: “... see wife, spouse’s, girlfriend, boyfriend, whatever’s notes”.’

(GP 31, male, 45–54 years)

*‘It’s* [the DVA] *alleged, I wouldn’t put anything in there* [the perpetrator’s EPR].’

(GP 19, male, 25–34 years)

These clinicians would only record in other family members’ EPRs if they had corroborating evidence of a child safeguarding issue:

*‘Certainly if there is documented evidence that there is a* [safeguarding] *case, it’s very difficult when there’s a suspicion, not a case.’*

(GP 15, female, age not given)

#### Continuity of care

Many of the clinicians described using the EPR to aid relational continuity of care (sometimes using code words to remind themselves of patient history), or informational continuity of care (often using free-text notes in case the patient saw a different clinician):

‘If I suspected I sometimes sort of do put something in the notes as a reminder to myself, I’ll perhaps put three question marks besides, you know, marital problems ??? … as a little remind to myself about them.’

(GP 41, female, 55–64 years)

‘It would be … about the level of domestic abuse, some key features, what the patient said, what the patient’s problems are, what their outcomes that they want, what the plan is for the action … so if anybody does read it, I’m off for a weekend or off ill or off on holiday, somebody else can pick up the case and coordinate.’

(GP 40, male, 45–54 years)

#### Information sharing for safety

Nearly all clinicians recognised that DVA was a child safeguarding issue and as such should be recorded in the children’s EPR (although some clinicians required ‘evidence’ to do so). Clinicians discussed documenting DVA as a first step in information sharing to highlight child safeguarding issues within the practice team, recognising other clinicians might see different family members:

‘The difficulty with safeguarding is, is making sure that, you know, information is there so that people can access it … so that no matter who goes into the records people know that there are safeguarding issues, because that’s ultimately what our job is as GPs, is to safeguard the children and the family and obviously the mum of the children.’

(GP 21, female, 45–54 years)

Just under half (*n* = 26) of the clinicians identified the importance of documenting DVA in children’s EPRs. A further 13 clinicians said they would also document in the perpetrator’s EPR. GPs discussed sharing this information to address related behaviours in the perpetrator such as mental health or alcohol consumption, or to provide an opportunity to challenge behaviour:

*‘If we’re aware of it* [DVA] *on his record then next time he came in about his drinking you could, well we would ask, you know, what kind of problems is this having, are you having any problems in your relationship, those types of things?’*

(GP 22, female, 55–64 years)

‘In some ways it’s quite a useful challenge to say “well look, you know, we’ve been advised about this issue, we record it on all the patients’ records because that is our practice” by way of communication and if he didn’t like it that’s his problem … Making him aware that we’re all aware and that he’s not going to get away with it.’

(GP 12, male, 55–64 years)

Thirteen clinicians explicitly stated, however, they would not document DVA in the perpetrator’s EPR. For some, this was because it was ‘only’ an allegation (see above), but for many it was because of concerns about escalating abuse if the perpetrator discovered the disclosure in their EPR:

*‘I wouldn’t* [document in perpetrator’s EPR] *because that partner* [the perpetrator] *has, has access to the notes* [has the right to view his EPR] *and, and the, the woman hasn’t actually given you permission to do that, and that could cause, you know, could cause lots of problems and could put her at more risk.’*

(GP 28, female, 55–64 years)

#### A patient-owned record

No clinician discussed the EPR as a mechanism to empower patients or families experiencing DVA. Clinicians were aware that patients could request to see their EPR or their children’s EPR. However, patient ownership and control of what is recorded was a difficult process:

*‘* [If] *she doesn’t want me to record it, I don’t record it because obviously she would not want me … that’s a very difficult situation, I can do all in my power to try and persuade her, I can give the statistics how if it’s physical but a lot of people do end up losing their lives … If the children are affected I can actually override her I feel and contact the child safeguarding teams. If there are no children involved and it’s just a woman, I, I don’t think I can override her wishes unless she had a mental health problem and I felt she could be sectioned but I can’t really, all I, it’s very difficult.’*

(GP 41, male, 45–54 years)

In contrast, another GP was aware that patients can access their notes, but this did not affect her decision to document as she felt the EPR is the property of the NHS:

*‘You know, my view is that the patient record is, it doesn’t really belong to them* [the patient]*, it belongs to the NHS and so it’s still OK for me to document that stuff and if they* [the perpetrator] *look, well that’s one of those things isn’t it? You know, it may offend them but if it’s honest and factual then there isn’t really a lot that they can do in terms of arguing, arguing over it.’*

(GP 03, female, 35–44 years)

Three GPs felt that patient access to online records was not an issue as the EPR represents a ‘*factual*’ (GP13, female, 45–54 years) account. Five were extremely concerned, however, that patient ownership of their own records actually increased the potential for a coercive partner to get access to the abused partner’s EPR:

‘It is difficult because, you know, in theory you would only have access to your own information or, or whatever; however, if you’re in a controlling relationship, you know, it’s not going to be very difficult for somebody inappropriate to get hold of a, you know, your login details or whatever, so I certainly see that as a, a risk to individuals.’

(GP 36, female, 45–54 years)

### Tensions among the different roles of the EPR

Multiple roles of the EPR resulted in uncertainty regarding the best way to document. The main tension identified was that between confidentiality to protect the abused parent and information sharing for child safety.

Clinicians were worried that the EPR might be seen accidentally by the perpetrator, resulting in an escalation of DVA for both the abused parent and children:

*‘We think there’s a danger in them* [the perpetrator] *accessing those records and, and thereby making the victims more vulnerable.’*

(GP 36, female, 45–54 years)

This led some clinicians to develop strategies to hide DVA documentation using code words and linking records using numbers:

*‘What we would do is we would put it on* [the perpetrator’s EPR] *as a “cause for concern”* [code] *but what we’d often do, simply because sometimes patients can be left in a room with a computer on and their records up, would be to* [write] *“See record* [and then give a record number]*”. So you’d just put like a “cause for concern” code on and then you’d perhaps put a code through to Sarah’s records rather than putting everything down.’*

(GP 22, female, 55–64 years)

Clinicians’ concerns about confidentiality increased as the number of family members involved multiplied.

Another tension occurred regarding the clinicians’ uses of the EPR versus the patients’ access to the EPR:

‘I think we write some things in some people’s records that they shouldn’t read and that’s on a very individual basis and … most people can read their records but I think in some situations they, there’s some things people shouldn’t read, and we’re putting it on there to warn other people and, and that’s to the benefit of the patient.’

(GP 10, male, 35–44 years)

Therefore, some clinicians discussed sharing information while avoiding documenting in the notes by sending e-mails or private messages within the practice:

‘I don’t think I’d put anything on the patient’s records but I would discuss with the other clinical staff about the situation, so just let them know or, so that would probably consist of sending an e-mail round on our Outlook NHS e-mail or internal e-mail to the other clinicians, saying please could you look out for this patient’s notes?’

(GP 33, female, 25–34 years)

## DISCUSSION

### Summary

There is substantial variation and inconsistency in general practice clinicians’ documenting of DVA in families with children. For a minority of clinicians, this is because of a lack of knowledge and understanding regarding DVA. However, the majority of clinicians recognised DVA and its impact on child safeguarding, but struggled to work out the best way to document it. There was a tension between managing the confidentiality of the victim alongside the safety of the children. On the one hand, it was acknowledged that safety could be improved through continuity of care and information sharing. On the other hand, most clinicians recognised that abuse could escalate if the perpetrator discovered disclosure. In addition, clinicians were managing information governance requirements and legal concerns about full and accurate records. Clinicians also recognised patients’ rights to view their own records, with concerns about the consequences of patients accessing information about DVA. This led individual practices, and in many cases individual clinicians on an *ad hoc* basis, to develop complex mechanisms and strategies to hide information so that it was only available to other clinicians.

### Strengths and limitations

Focusing on how general practice clinicians document DVA in families illuminates the complexity and tensions between competing demands on the clinician. The intensive and wide-ranging recruitment strategy led to a large and varied sample of clinicians, resulting in thematic saturation despite the complexity of the topic area. Many responders did not have experience of domestic violence cases or training, reflecting the reality of general practice. The vignette allowed participants with little experience to discuss the topic; however, what clinicians say in response to a vignette may differ from their practice. Health visitors were not included in the present sample as the focus was on general practice. However, many of the responders discussed the role of health visitors in managing DVA in families with children (results reported elsewhere).^27^

### Comparison with existing literature

Hester described three distinct policy ‘planets’ that exert influence on the management of DVA in families with children: care and support for the victim; care and support for the children; and care and support for the abusive parent.^28^ Focusing on documenting in general practice demonstrates that clinicians are influenced by each of these competing conceptualisations of DVA. They describe documenting to provide continuity of care for the victim, to protect children, and as an opportunity to address the perpetrator’s behaviour. However general practice clinicians are being influenced by, and need to respond in all three policy directions simultaneously, as well as non-DVA policies of information governance and patient access to records.

Previous research demonstrates that general practice professionals respond to different national policies by adapting them to local priorities, workloads, and resources.^29^^,^^30^ In the absence of national and local guidance on how to document DVA in families, clinicians are developing their own often intricate methods of documenting based on their expectations of the role of the EPR. This is complicated by the development of separate mandatory training and documenting advice for each role of the EPR, developed in silos. This may explain some of the inconsistent documenting strategies, as individual clinicians in individual practices try to balance the competing ‘planets’ and make sense of their work. Similar inconsistencies have been observed in general practice clinicians’ documenting of male involvement in DVA (E. Williamson, personal communication, 2016). This suggests the need for a wider, cohesive discussion of the role of the EPR.

With the advance of new technology, the EPR is increasingly seen as having a wide range of unforeseen effects.^13^ The effect of the computer and more recently the EPR on the consultation in general practice has been described previously.^20^^,^^31^^,^^32^ Creswell and colleagues describe the EPR as an actor: *‘... the source of an action regardless of its status as a human or non-human’*.^33^ The EPR is one actor in a network with other human (clinician) and non-human (policies) actors. The interactions between the different actors shape the resulting action.^33^ In this study clinicians, acting in a network with child safeguarding policy and the EPR, produce the action of information sharing. Creswell and colleagues found that the EPR can have different roles, with different meanings for different stakeholders depending on the context.^33^ Therefore in response to the same stimulus (the vignette), different clinicians form different connections with each policy ‘planet’ and the EPR, to produce different actions and concerns. Adding in more actors, such as patients with online access, makes the networks more complex, resulting in unpredictable outcomes that can be different from those initially desired.^33^ In the present study this resulted in clinician uncertainty: will documenting to share information result in the information being shared with the intended people, or unintended disclosure to a perpetrator and reduced safety for victim and children? The tensions described by clinicians result from both the complexity of DVA in families, and the uncertainty of working with a network of actors, including the EPR, policies, and patients, where actions may have unforeseen consequences. Online access to the EPR may become a tool of coercion for patients experiencing DVA; this has not been adequately addressed in terms of policy or technical solutions for hiding sensitive information.^22^

### Implications for practice

Managing DVA in families with children is a challenge in general practice. Focusing on how clinicians document DVA in the EPR demonstrates the competing policy priorities acting on clinicians and the multiple potential actions triggered by documenting. Not only are clinicians being asked to care and support all members of the family, but they are also being asked to provide a legal record and a record for patient use. In the absence of national and local guidance, clinicians are developing individual strategies to balance these competing priorities. This is resulting in inconsistent and confused documenting strategies. There is a need for national integrated guidance on documenting taking into account all actions of the EPR. This should be supported by mandatory training covering how to respond to the female,^5^ children,^34^ and male^35^ victims. This is even more urgent given the move to online EPRs with patient access.^22^ This has wider implications for documenting sensitive information in general. Further access to patient online records should not be made available without providing national safeguards and guidance about how clinicians can use the EPR safely to provide a legal record, continuity of care to vulnerable groups, and to share child safeguarding information. Potential solutions include flexibility within the EPR to have private and shared spaces, but this would require public agreement regarding the ownership of and access to their data. This needs to be done following a national conversation involving the public (both those with experience of DVA and those without), general practice clinicians, IT developers, and policymakers.
